# Cyclic Mechanical Loading of Cardiomyocytes via Pressure‐Driven Non‐Planar Membrane Deformation in a Bioreactor System

**DOI:** 10.1002/advs.202523820

**Published:** 2026-07-13

**Authors:** Haris Mansoor, Gabrielle Juul, Jil Patel, Heran Pradhan, Gram Hepner, Jackson Jewell, Mark Bardin, Ryan Slusser, Abigail Glezer, Leda Klouda, Melikhan Tanyeri, Anita Saraf

**Affiliations:** ^1^ Heart Institute, Department of Pediatrics UPMC Children's Hospital of Pittsburgh Pittsburgh Pennsylvania USA; ^2^ Department of Medicine UPMC Heart and Vascular Institute Pittsburgh Pennsylvania USA; ^3^ Department of Biomedical Engineering, School of Science and Engineering Duquesne University Pittsburgh Pennsylvania USA; ^4^ Department of Pediatrics RK Mellon Institute for Pediatric Research Pittsburgh Pennsylvania USA

**Keywords:** cardiac tissue engineering, cardiomyocyte maturation, cyclic mechanical preload, heart‐on‐a‐chip, hiPSC‐CMs

## Abstract

We introduce a novel bioreactor system that applies cyclic strain through controlled non‐planar membrane deformation. The membrane deformation is driven by tunable pressure profiles spanning clinically reported end‐diastolic pressure ranges observed in physiological and disease‐associated conditions. Applied pressure was quantitatively related to spatial membrane strain through three‐dimensional membrane reconstruction, enabling characterization of the mechanical environment at the cell–substrate interface. Human induced pluripotent stem cell‐derived cardiomyocytes (hiPSC‐CMs) are cyclically loaded for three days and analyzed for structural, functional, and transcriptomic responses. The bioreactor system preserves cellular viability under pressures ranging from 0–15 mmHg. Cells exposed to physiological preloads (5 and 10 mmHg) exhibit functional and transcriptional changes associated with cardiomyocyte maturation, including cell elongation and altered contractile waveform kinetics, while those under pathological preloading conditions (15 mmHg) show activation of stress‐related and catabolic signaling pathways commonly reported in mechanical overload and heart failure models. This platform enables precise modeling of myocardial biomechanics and offers a robust tool for investigating cardiomyocyte response across health and disease states.

## Graphical abstract

In this research article, Mansoor and coworkers present a novel device that applies controlled mechanical stimuli to cardiomyocytes using pressure‐driven membrane deformation. By exposing cells to physiologically and pathologically relevant loading conditions, the platform reproduces distinct structural, functional and molecular responses associated with cardiac adaptation and mechanical stress, providing a versatile model to study cardiac mechanobiology in vitro.

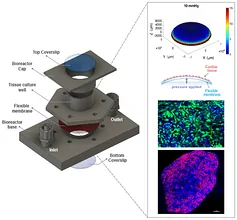

## 1 Introduction

Cardiomyocytes (CMs) impart beating function to the myocardium. These cells contract continuously from their emergence in the embryonic heart tube, coupling systemic perfusion with intrinsic sensitivity to mechanical forces. During development and postnatal conditions, venous filling directs passive myocardial stretch in a longitudinal, circumferential, and radial direction, termed as preloading [1, 2]. Clinically, preload is estimated by measuring end‐diastolic pressure, which reflects the passive filling forces acting on the myocardium prior to systole [3]. In the human left ventricle, end‐diastolic pressures average 3–7 mmHg during late fetal development, and increase to approximately 5–10 mmHg postnatally and into adulthood [4, 5, 6, 7].

As the heart undergoes morphological transformation from a cylindrical heart tube to a complex four‐chamber pump with vascular connections for inflow and outflow of blood, cardiomyocytes concurrently mature into elongated, anisotropic, and electromechanically connected structures, with organized sarcomeres that drive contractility. In the postnatal period, cardiopulmonary adaptation leads cardiomyocytes to a hypertrophic growth pattern, accompanied by a cascade of biochemical and structural remodeling events to enhance contractile strength and cardiac output [8]. Pathological conditions, including those emerging in utero, can disrupt this process, leading to altered cardiomyocyte structure and function [8, 9]. In disease states, maladaptive remodeling of the myocardium leads to a loss of cellular organization, synchronized contractility, and intercellular communication. Clinically, loss of contractile function in conditions such as dilated cardiomyopathy results in elevated preload pressures [10].

Despite significant advances in understanding the molecular pathways regulating cardiac development and remodeling, existing in vitro systems remain limited in their ability to replicate physiologically relevant biomechanical cues. At the tissue level, cardiomyocytes experience cyclic multiaxial mechanical strain due to rhythmic myocardial contraction and relaxation. Various bioreactor systems have been developed to model aspects of the cardiac beating cycle, including planar stretch platforms [11, 12, 13] and heart‐on‐chip devices [14, 15, 16, 17, 18]. These systems typically apply uniaxial or biaxial strain within two‐dimensional configurations, defining mechanical inputs in terms of imposed strain or actuation parameters rather than physiologically relevant preload pressures. Although pneumatic or vacuum pressure is often used to generate strain, the applied pressure values typically serve as engineering control parameters selected to achieve a target deformation rather than representing clinically interpretable end‐diastolic preload pressures [11, 13, 16, 19, 20]. Engineered heart tissues (EHTs) [21, 22, 23] and microtissue constructs provide higher‐order structural organization and tissue‐level loading; however, preload is not consistently anchored to defined pressure values, and quantitative reconstruction of spatial strain fields is often not performed [11, 24]. Consequently, the quantitative relationship between the applied actuation input and the resulting spatial strain experienced at the cell–substrate interface remains incompletely characterized in many existing systems.

In this study, we present a novel bioreactor platform that applies cyclic pressure profiles simulating end‐diastolic pressures in order to induce membrane deformation, generating non‐planar, multiaxial strain fields at the cardiomyocyte–substrate interface. Using three‐dimensional membrane reconstruction by fiduciary marker tracking, we quantitatively map applied pressure to in‐plane membrane strain. By anchoring mechanical stimulation to preload pressures (5, 10, and 15 mmHg) observed across late fetal/early postnatal, adult, and pathological conditions, respectively, the platform establishes a defined mechanical link between physiologically relevant filling pressures and the cellular loading state, while maintaining a monolayer culture format compatible with imaging and downstream molecular assays.

To evaluate the biological relevance of this platform, human iPSC‐derived cardiomyocytes (hiPSC‐CMs) were cultured on deformable membranes within the bioreactor chamber. Human iPSC‐CMs were chosen for their relevance to human physiology and disease modeling, despite their known structural and functional immaturity [25]. When combined with controlled mechanical conditioning and phenotypic characterization, these cells provide a valuable model to investigate mechanobiological responses under defined preload conditions [26, 27, 28, 29, 30]. We hypothesized that controlled, pressure‐calibrated preload would induce measurable structural, functional, and transcriptional adaptations consistent with mechanosensitive remodeling. We therefore exposed hiPSC‐CMs to cyclic preload pressures ranging from 0–15 mmHg over a 3‐day period to evaluate effects on viability, structural organization, contractile function, and transcriptomic remodeling.

## 2 Results

### 2.1 Bioreactor Design, Fabrication, and Characterization

We designed and fabricated a custom bioreactor to replicate physiologically relevant mechanical loading conditions experienced by myocardial tissue during cyclic filling. The bioreactor consists of two chambers separated by a thin, flexible membrane that serves as the substrate for cardiomyocyte culture, providing a total culture surface area of approximately 2.5 cm^2^ (Figure 1A). The upper chamber provides a controlled environment for cell growth while the lower chamber is pneumatically actuated to deform the membrane, producing cyclic mechanical strains that mimic the dynamic forces experienced in the native myocardium. By modulating pressure in the lower chamber, we can precisely control both the magnitude and frequency of membrane deformation, enabling tunable cyclic strain application.

**FIGURE 1:**
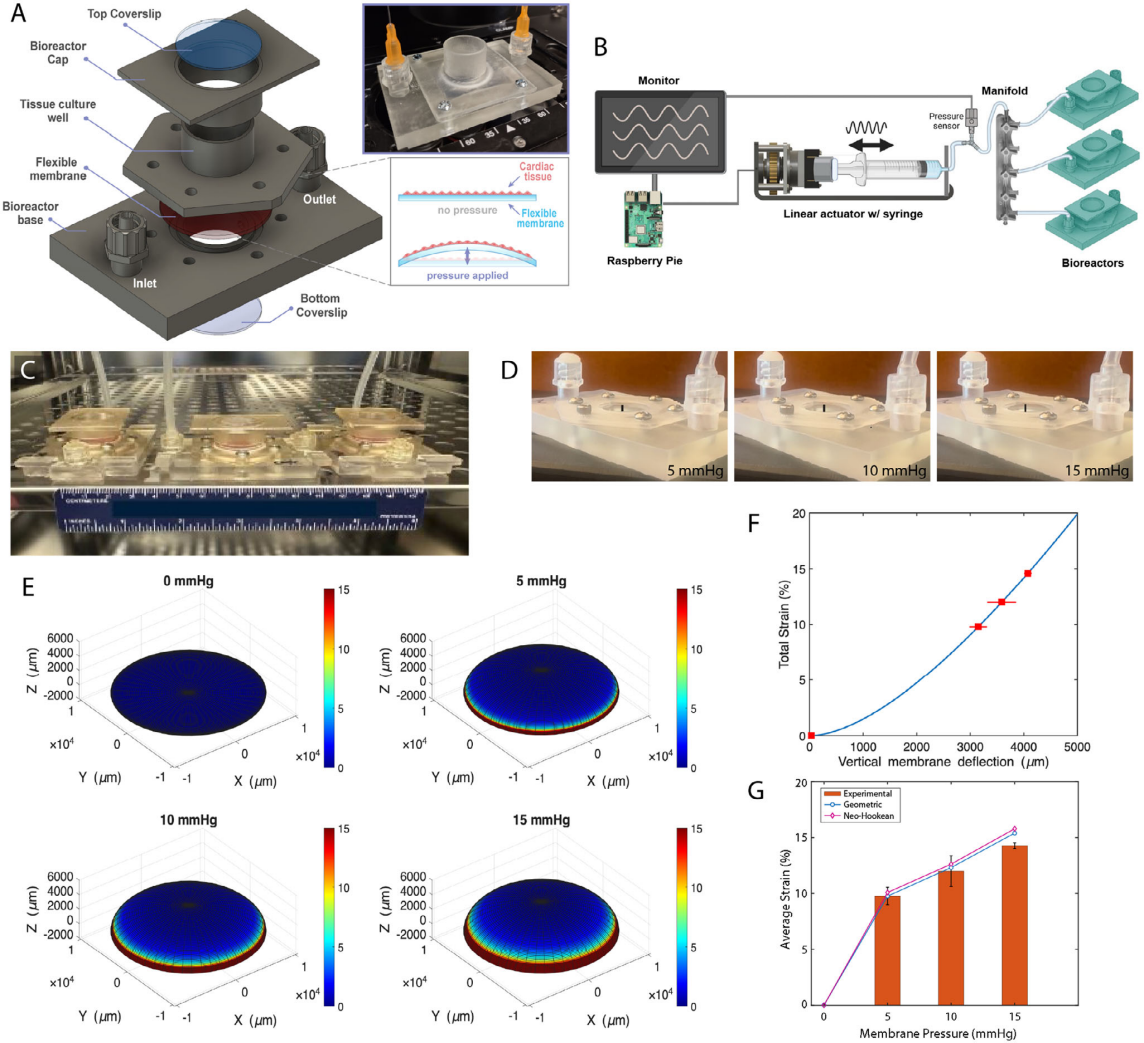
Multi‐chamber bioreactor system for mechanostimulation of cardiomyocytes. (A) Schematic of components and assembly of the cardiac bioreactor (left). The bioreactor prototype was assembled using multiple components fabricated via 3D stereolithographic printing (top right). 3D deformation of membrane emulates ventricular displacement with each cardiac cycle (bottom right). (B) Schematic of experimental setup for the multi‐bioreactor system including custom pneumatic actuation system, Raspberry Pi microcontroller, pressure sensor, and connected bioreactors. (C) Multiple bioreactor units can be isolated from the pneumatic actuation system inside an incubator to preserve sterile conditions. Ruler included for scale. (D) Images demonstrating vertical deflection of the silicone membrane under increasing applied pressures (5–15 mmHg). (E) Corresponding strain heat‐maps depicting spatial distribution of deformation across the membrane at each pressure value (0–15 mmHg). (F) Quantification of total strain across the membrane corresponding to the vertical deflections at each pressure. (G) Average membrane strain at each preloading condition. Membrane deflection and average strain are shown as mean ± SD (n = 3 independent deformation measurements).

To apply controlled cyclic strain, we developed a custom pneumatic actuation system integrated with the bioreactor platform (Figure 1B). The system uses a linear actuator coupled to a syringe and controlled by a Raspberry Pi to precisely modulate the amplitude and frequency of applied pressures. These pressures are delivered via a manifold to one or more bioreactors (Figure 1C), with a pressure sensor in series providing real‐time feedback to ensure precise control of loading conditions. While the system allows implementation of custom waveforms, we applied a sinusoidal waveform at 1 Hz to generate pressure profiles that approximate clinically relevant preload conditions. When pressure is applied to the reservoir beneath the flexible membrane, the membrane deflects upward into the culture chamber (Figure 1D), generating mechanical strain on the cardiomyocytes seeded on its surface. The magnitude of deflection, and thus the applied strain, is directly dependent on the applied pressure. By applying tunable cyclic strain through controlled out‐of‐plane membrane deflection, the bioreactor generates spatially heterogeneous, multiaxial strain fields across the membrane surface and offers a physiologically relevant model for studying how cardiomyocytes sense and adapt to mechanical forces. Integrated optical windows at the top and bottom of the bioreactor enable real‐time, high‐resolution imaging using brightfield, phase‐contrast, fluorescence, and confocal microscopy, facilitating continuous monitoring of cardiomyocyte behavior during mechanical stimulation.

We evaluated preload conditions spanning clinically reported end‐diastolic pressures, including a no‐load control (0 mmHg), low‐pressure loading (5 mmHg), adult physiological preload (10 mmHg), and elevated filling pressures (15 mmHg) associated with pathological states. To quantify the relationship between applied pressure and resulting membrane strain, we reconstructed the three‐dimensional membrane geometry at each pressure (0–15 mmHg) using fiduciary marker tracking and generated strain heatmaps across the surface (Figure 1E). The strain heatmaps exhibit radial symmetry consistent with dome‐shaped axial deflection. The reconstructed three‐dimensional geometry also captures membrane curvature, which underlies the observed spatial strain gradients across the surface. Strain calculations assumed primarily axial (out‐of‐plane) deformation, an assumption supported by fiduciary marker analysis showing negligible lateral displacement. From the reconstructed geometry, spatial strain fields were computed and total membrane strain quantified (Figure 1F). Across three independent trials (*n* = 3), average membrane strain increased with applied pressure: 0% at 0 mmHg, 9.77 ± 0.78% at 5 mmHg, 12 ± 1.37% at 10 mmHg, and 14.47 ± 0.15% at 15 mmHg (Figure 1G).

To further validate the pressure–strain relationship, we developed a complementary mechanical model of the deflected membrane (Section S3). Using experimentally measured center deflection as input, line‐averaged engineering strain was computed using geometric and Neo‐Hookean finite‐deformation formulations. Predicted strains deviated from experimentally reconstructed values by less than ≈1%–1.5% absolute strain across the 5–15 mmHg range, indicating close quantitative agreement between experimental reconstruction and continuum‐based modeling. These results confirm that membrane deformation in this regime is governed primarily by geometric nonlinear effects associated with large deflection. Although cardiomyocytes are cultured as adherent monolayers on a two‐dimensional substrate, cyclic pneumatic actuation induces non‐planar membrane deformation that generates spatially heterogeneous, multiaxial strain fields, such that in‐plane membrane strain provides a physically grounded proxy for the mechanical loading experienced at the cell–substrate interface.

### 2.2 Bioreactors Preserve hiPSC‐CM Viability

Cardiomyocytes were differentiated from human induced pluripotent stem cells (hiPSCs) using an established protocol [31] (Figure 2A), achieving a differentiation efficiency of 78.9 ± 3.7%, as determined by NKX2.5 immunostaining. Bioreactors were assembled and differentiated cardiomyocytes (hiPSC‐CMs) were seeded onto the silicone membrane on day 15. After 48 h of incubation to promote cell adhesion, cardiomyocyte cultures were subjected to cyclic strain for 3 h daily over 3 days, followed by 24 h of rest‐period before membrane extraction and analysis (Figure 2B). Brightfield imaging confirmed the formation of a continuous, adherent cardiomyocyte layer across the membrane surface over a 72‐hour period after initial seeding (Figure 2C). Control experiments performed in the absence of cyclic strain demonstrated sustained viability, as assessed by live/dead staining (Figure 2C). Quantitative analysis revealed no statistically significant differences in viability over time: 86.6 ± 3.8% at 24 h, 88.3 ± 3.5% at 48 h, and 83.5 ± 2.1% at 72 h (*p* = 0.08; Figure 2D), indicating that the bioreactor environment maintained hiPSC‐CM viability in the absence of mechanical stimulation.

**FIGURE 2:**
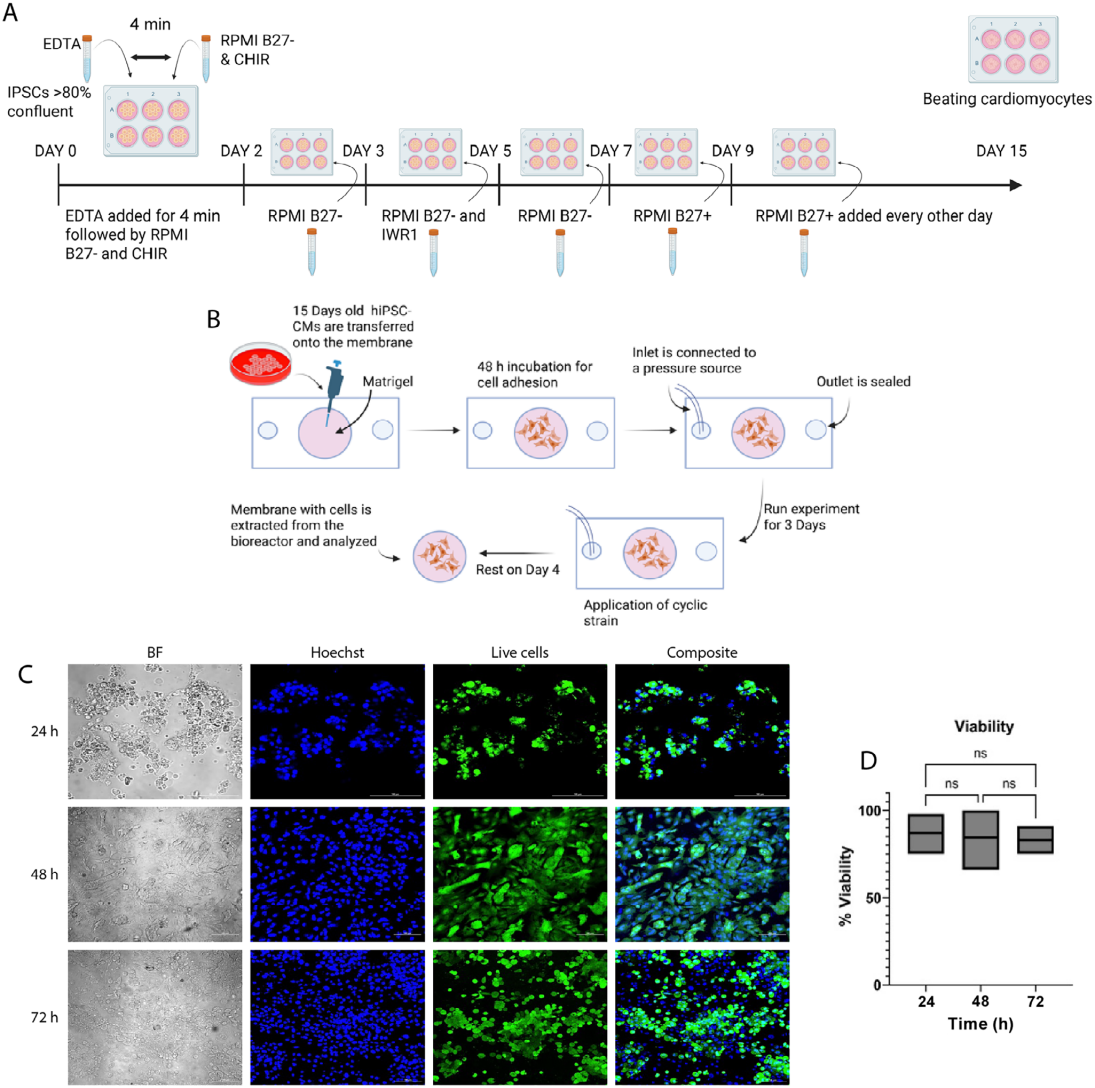
hiPSC‐CMs seeded onto the bioreactor showed preserved cell viability. (A) Schematic outlining the protocol for differentiating hiPSCs into beating cardiomyocytes. (B) Schematic of the experimental workflow showing hiPSC‐CMs seeded onto bioreactors, exposed to cyclic preload pressures, and harvested for downstream analysis. (C) Cell viability assay of hiPSC‐CMs under static (no‐strain) conditions at 24 h, 48 and 72 h after seeding onto the bioreactor membrane. BF: brightfield. Scale bar: 100 µm D) hiPSC‐CMs viability remained stable at 24 h, 48 h, and 72 h post‐seeding as quantified by cell viability assay. Data are presented from three independent biological replicates (N = 3). No significant differences in viability were observed across time points (one‐way ANOVA with Tukey's multiple‐comparison test. ns, not significant; *p* > 0.05).

### 2.3 Preload‐Dependent Changes in hiPSC‐CM Viability, Morphology, and Structural Maturation Associated Features

To assess the effects of cyclic preload on hiPSC‐CM viability, cardiomyocyte cultures were subjected to increasing levels of cyclic strain induced by membrane pressures ranging within 0–15 mmHg (Figure 3A). Brightfield and live/dead fluorescence imaging revealed increased cell coverage across the membrane and a mild decrease in cell‐viability under moderate preload conditions (5–10 mmHg), with a marked decline at high preload conditions (15 mmHg). Quantitative analysis confirmed that viability remained relatively stable after 3 days of cyclic strain at 5 mmHg (82.4 ± 1.7%, *p* = 0.0346) and 10 mmHg (81.0 ± 1.7%, *p* = 0.0009) but decreased significantly at 15 mmHg (75.4 ± 3.5%, *p* < 0.0001) compared to the no‐strain control (Figure 3B).

**FIGURE 3:**
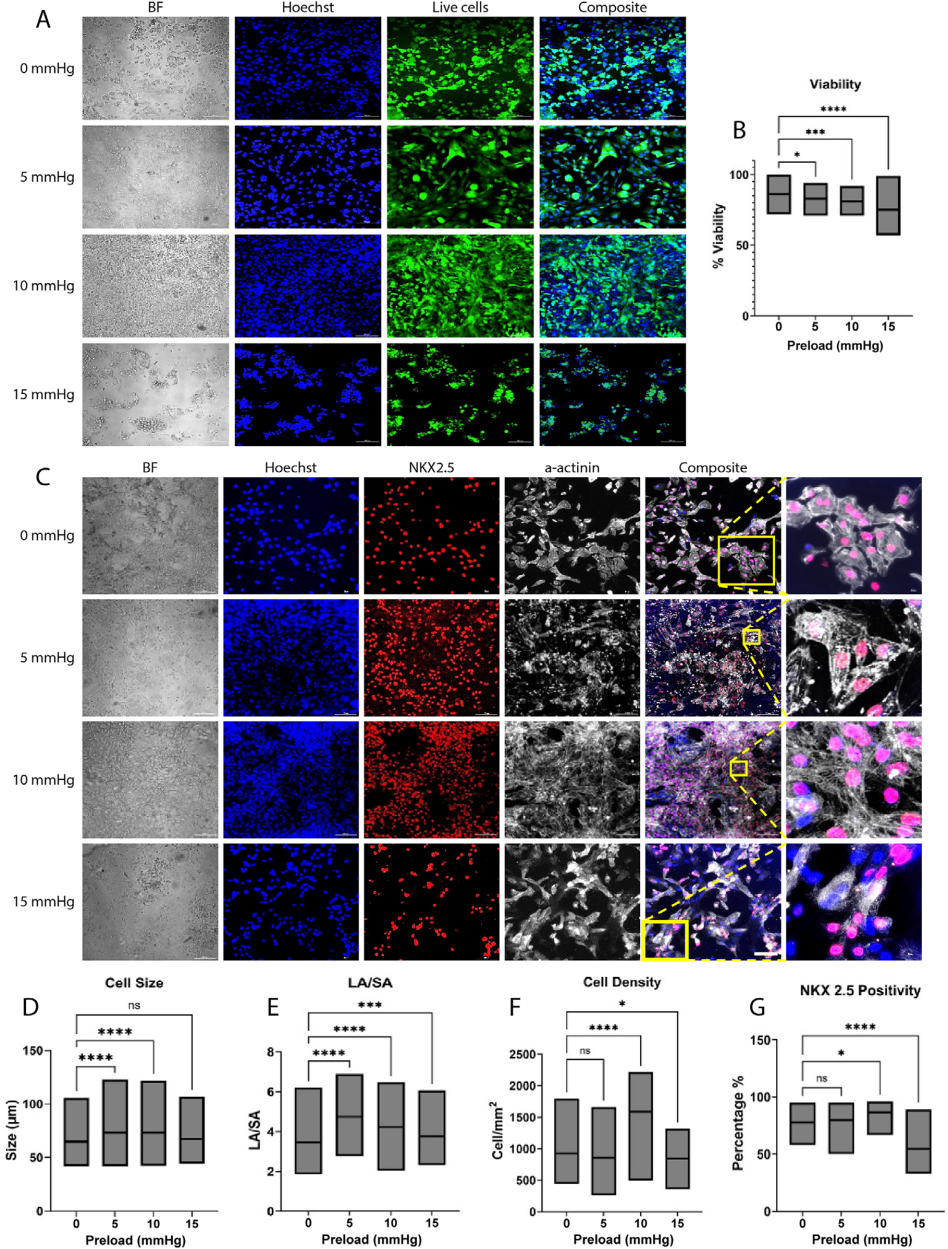
hiPSC‐CMs subjected to cyclic preload showed changes in viability and cellular characteristics. (A) Viability assay for hiPSC‐CMs after 3 days of cyclic preloading in the bioreactor. (B) Quantitative analysis shows that viability remained relatively stable under moderate preload (5–10 mmHg) while decreasing at high preload (15 mmHg). (C) Immunofluorescence staining of hiPSC‐CMs for Hoechst (blue), NKX2.5 (red) and α‐actinin (white) at 0, 5, 10, and 15 mmHg preloading conditions. (D) Cell size slightly increased under moderate cyclic preload (5–10 mmHg), but remained unchanged at high preload (15 mmHg). (E) LA/SA ratio, indicative of cell elongation, increased at 5 and 10 mmHg but decreased at 15 mmHg compared to static conditions (0 mmHg). (F) Cell density significantly increased at 10 mmHg and decreased at 15 mmHg. (G) NKX 2.5 expression increased at 10 mmHg but decreased significantly at 15 mmHg. Scale bar: 100 µm, BF: Brightfield. Data are presented from three independent biological replicates (N = 3). Statistical significance was determined using one‐way ANOVA with Tukey's multiple comparison test. ns, not significant; ^*^ *p* < 0.05, ^***^ *p* < 0.001, and ^****^ *p* < 0.0001.

Compared to static conditions (0 mmHg), cardiomyocyte size increased under moderate cyclic preload (5–10 mmHg) but remained unchanged at high preload (15 mmHg). Brightfield imaging, nuclear staining, and NKX2.5 immunolabeling revealed increasing cardiomyocyte density across the membrane from 0 to 10 mmHg (Figure 3C). Cell size analysis showed a preload‐dependent increase in long‐axis (LA) length: 65.2 ± 1.2 µm at 0 mmHg, 74.0 ± 1.7 µm at 5 mmHg, and 76.0 ± 1.8 µm at 10 mmHg (*p* < 0.0001), followed by a decline to 67.1 ± 1.2 µm at 15 mmHg (*p* = 0.2089, Figure 3D). Additionally, α‐actinin staining showed preload‐dependent sarcomeric organization (Figure S1). hiPSC‐CMs under static conditions (0 mmHg) appeared small, round, and displayed disorganized sarcomeres. In contrast, cells exposed to 5 and 10 mmHg showed increased elongation with anisotropic alignment and well‐defined Z‐bands, indicating organized sarcomeric architecture. At 15 mmHg, structural organization was disrupted and cell size decreased. These results suggest that physiological preload promotes cardiomyocyte elongation and sarcomeric organization consistent with early maturation‐associated changes, while excessive mechanical loading impairs structural integrity.

The LA/SA ratio, a measure of cardiomyocyte elongation, increased under moderate cyclic preload but declined at higher levels. Immature hiPSC‐CMs are typically round with LA/SA ratios ranging from 1–2, while adult human cardiomyocytes exhibit higher elongation, with LA/SA ratios in the range of ≈5–9 [32]. In our study, cells under static conditions (0 mmHg) had a LA/SA ratio of 3.6 ± 0.2. This increased to 4.7 ± 0.1 at 5 mmHg and 4.4 ± 0.1 at 10 mmHg (*p* < 0.0001), indicating increased elongation. However, at 15 mmHg, the ratio decreased to 3.8 ± 0.1 (*p* < 0.0001), reflecting a loss of elongated morphology (Figure 3E). These values are consistent with partial maturation of hiPSC‐CMs, which remain less elongated than adult cardiomyocytes.

Cell density, measured as DAPI‐stained nuclei per unit area, remained statistically unchanged at 5 mmHg (895 ± 45 cells mm^−2^; *p* = 0.0848) compared to baseline at 0 mmHg (983 ± 55 cells mm^−2^). However, a significant increase was observed at 10 mmHg (1504 ± 83 cells mm^−2^; *p* < 0.0001), indicating enhanced cell survival or retention under moderate cyclic preload. In contrast, cell density markedly declined at 15 mmHg (841 ± 34 cells mm^−2^; *p* = 0.0015), suggesting that excessive mechanical strain may impair viability (Figure 3F).

Expression of NKX2.5, a CM‐specific nuclear transcription factor, remained stable at 5 mmHg (76 ± 6.1%, *p* = 0.7) relative to control levels at 0 mmHg (78.9 ± 3.8%), indicating preserved differentiation (Figure 3G). At 10 mmHg, NKX2.5 expression increased significantly to 85.6 ± 2.3% (*p* = 0.0497), suggesting enhanced cardiac differentiation under moderate cyclic preload. However, at 15 mmHg, expression decreased markedly to 57.9 ± 7.5% (*p* < 0.0001), indicating a loss of cardiomyocyte identity.

Overall, our findings indicate that moderate cyclic preload (5–10 mmHg) promotes early maturation‐associated structural and molecular adaptations in hiPSC‐CMs, reflected by increased cell density, elongation, anisotropic sarcomere organization, and elevated NKX2.5 expression (Figure 3D–G). However, pathological preload (15 mmHg) led to reduced cell viability and density, loss of molecular and structural markers of maturation; features consistent with impaired differentiation. These results suggest a threshold beyond which preload becomes detrimental to cardiomyocyte development and function.

### 2.4 Cyclic Preload Modulates Cnx43 Expression and Localization in a Load‐Dependent Manner

Connexin 43 (Cnx43), a gap junction protein critical for intercellular Ca^+2^ signaling, is upregulated during CM maturation and downregulated in myocardial disease [33, 34]. In our studies, Cnx43 expression increased under moderate preload (0–10 mmHg), reflecting enhanced intercellular connectivity, but decreased significantly at pathological levels (15 mmHg), akin to patterns observed in disease (Figure 4A). Z‐projected immunofluorescence images showed that Cnx43 distribution changed markedly with increasing preload. At 0–5 mmHg, Cnx43 was distributed both along cell‐cell junctions and within the cytoplasm. At 10 mmHg, Cnx43 expression became more localized at intercellular junctions, suggesting the formation of gap junctions. However, this localization was disrupted under pathological loading (15 mmHg) where Cnx43 localization shifted predominantly to cytoplasm.

**FIGURE 4:**
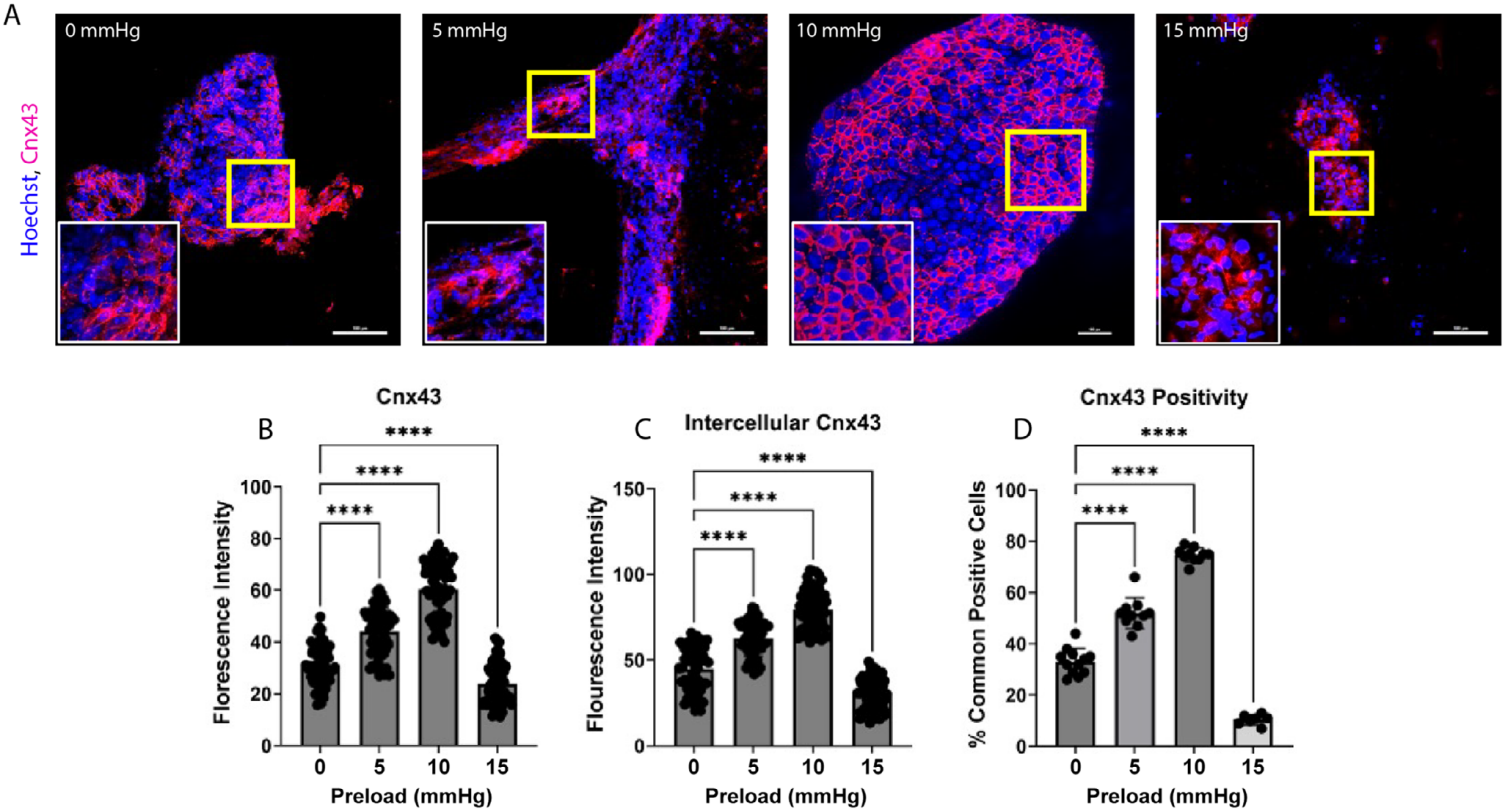
Connexin‐43 (Cnx43) expression and localization are modulated by cyclic preload. (A) Representative immunofluorescence images showing Cnx43 (red) and nuclei (blue) under increasing cyclic preload conditions (0–15 mmHg). Cnx43 expression increased, and its distribution shifted from diffuse to junctional under moderate preload (5‐10 mmHg), but became disrupted at high preload (15 mmHg). (B) Total Cnx43 fluorescence intensity increased significantly at 5 and 10 mmHg, peaking at 10 mmHg, but decreased at 15 mmHg. (C) The proportion of Cnx43 localized at cell‐cell junctions was highest at 10 mmHg and significantly reduced at 15 mmHg. (D) The percentage of Cnx43‐positive cells increased with physiological preload (5–10 mmHg) and declined at pathological levels (15 mmHg). Data are presented as mean ± SD (N = 3 independent biological replicates). Statistical significance was determined using one‐way ANOVA with Tukey's multiple comparison test. ^****^ *p* < 0.0001.

Quantitative analysis of total Cnx43 fluorescence intensity revealed a preload‐dependent response. Expression increased significantly from 31.4 ± 2.0 a.u. at 0 mmHg to 44.0 ± 2.4 and 60.3 ± 2.7 a.u. at 5 and 10 mmHg, respectively (*p* < 0.0001; Figure 4B). However, at 15 mmHg, total intensity decreased sharply to 23.8 ± 2.0 a.u. (*p* < 0.0001), consistent with impaired expression under excessive mechanical strain.

Additionally, we quantified the proportion of Cnx43 localized at cell–cell interfaces relative to total expression to assess intercellular localization. This intercellular fraction increased substantially from 45.9 ± 4.3% at baseline (0 mmHg) to 62.3 ± 3.0% and 80.4 ± 3.5% at 5 and 10 mmHg, respectively (*p* < 0.0001; Figure 4C). However, at 15 mmHg, this value significantly decreased to 31.4 ± 3.0% (*p* < 0.0001), indicating disrupted gap junction formation and a loss of connectivity under pathological loading. Similarly, the percentage of hiPSC‐CMs expressing Cnx43 between adjacent cells also rose from 33.1 ± 3.2% at no preload to 52 ± 4.3% and 74.7 ± 4% at 5 and 10 mmHg (*p* < 0.0001; Figure 4D). At 15 mmHg, this expression dropped dramatically to 10.4 ± 1.8% (*p* < 0.0001), further supporting a loss of intercellular connectivity and downregulation of gap junctional communication in response to excessive mechanical stress.

### 2.5 Mechanical Preload Modulates Cardiomyocyte Contractility, Beating Dynamics, and Waveform Kinetics

Contractility was analyzed under varying preload conditions using Myocyter analysis of brightfield video microscopy [35]. As hiPSC‐CMs mature, their spontaneous beating frequency and variability decrease [20, 36]. Spontaneous beating frequency was 0.6 ± 0.05 Hz in the absence of preload (0 mmHg), significantly decreased to 0.2 ± 0.07 Hz at 10 mmHg (*p* < 0.0001), then subsequently increased to 0.9 ± 0.2 Hz at 15 mmHg (*p* < 0.0001), suggesting altered spontaneous contractile dynamics under pathological load (Figure 5A). Contraction amplitude, a measure of contractile strength, increased from 21.4 ± 9.5 (a.u.) at 0 mmHg to 32.4 ± 12.0 (a.u.) at 10 mmHg (*p* < 0.0001), consistent with a preload‐dependent enhancement of contractile amplitude, but markedly decreased to 5.8 ± 3.7 (a.u.) at 15 mmHg (*p* < 0.0001), indicating loss of contractile efficiency (Figure 5B).

**FIGURE 5:**
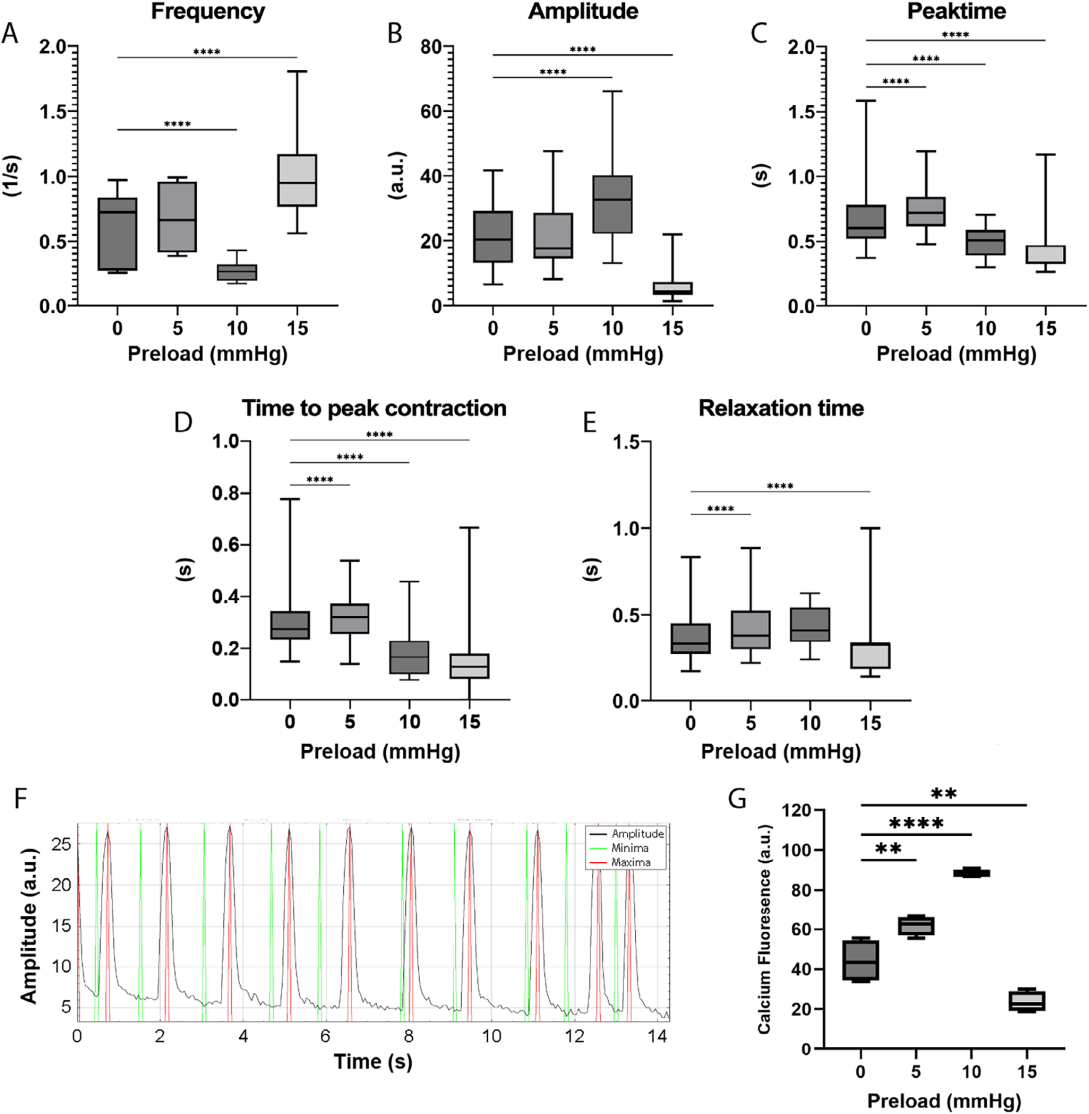
Cyclic preload modulates hiPSC‐CM contractility and calcium handling. Myocyter analysis of brightfield videos of spontaneously beating hiPSC‐CMs quantifying key dynamic parameters across 0–15 mmHg preload: (A) Beating frequency, (B) Contraction amplitude, (C) Peak time, (D) Time to peak contraction, (E) Relaxation time. (F) Representative contraction waveform of spontaneously beating cardiomyocytes at 10 mmHg. (G) Quantification of peak calcium transient intensity measured from calcium imaging following cyclic preload conditioning. Data are presented from three independent biological replicates (N = 3). Statistical significance was determined using one‐way ANOVA with Tukey's multiple comparison test. ^**^ *p* < 0.01, ^****^ *p* < 0.0001.

Temporal features of the contraction waveform, including peak time, time to peak contraction, and relaxation time collectively reflect the timing characteristics of each contraction cycle. These parameters describe waveform kinetics derived from spontaneous contraction traces obtained by brightfield video analysis of hiPSC‐CM monolayers. Shorter durations across these parameters indicate faster and more synchronized contractile behavior. Our data showed that temporal parameters of contraction were also modulated by preload. The peak time, defined as the width of the contractile waveform at 50% of peak height, remained relatively stable at 5 mmHg (0.7 ± 0.1s, *p* < 0.0001) compared to static conditions (0.7 ± 0.3s), but then decreased to 0.5 ± 0.01s at 10 mmHg and 0.4 ± 0.3s at 15 mmHg (*p* < 0.0001), indicating accelerated yet more variable contraction kinetics under high preload (Figure 5C). Time to peak contraction decreased from 0.33 ± 0.10s under no preload to 0.19 ± 0.11s at 10 mmHg and 0.15 ± 0.13s at 15 mmHg (*p* < 0.0001, Figure 5D). Relaxation time first increased modestly from 0.37 ± 0.15s (0 mmHg) to 0.41 ± 0.14s (5 mmHg) and 0.43 ± 0.12s (10 mmHg) but shortened to 0.32 ± 0.18s at 15 mmHg (*p* < 0.0001, Figure 5E). Variability in contraction and relaxation timing decreased from 0 to 10 mmHg but increased again at 15 mmHg. Figure 5F displays a representative contraction waveform at 10 mmHg, highlighting reduced peak width and lower beating frequency compared to static conditions (see also Figure S2). At 15 mmHg, contractions became irregular in amplitude, morphology, and frequency (Figure S2). Brightfield videos of spontaneously beating hiPSC‐CMs further demonstrate preload‐dependent changes in contraction amplitude, frequency, and waveform kinetics in response to varying levels of preload (Movies S1–S4). To further assess functional responses to preload, intracellular calcium dynamics were evaluated using calcium imaging. Peak calcium transient intensity exhibited a preload‐dependent response (Figure 5G), increasing from 44.1 ± 5.4 a.u. at 0 mmHg to 61.8 ± 2.4 a.u. at 5 mmHg (p < 0.01) and reaching a maximum of 88.2 ± 1.8 a.u. at 10 mmHg (p < 0.0001). In contrast, peak intensity decreased markedly at 15 mmHg to 23.5 ± 2.6 a.u. (p < 0.01). These findings indicate enhanced calcium handling under moderate preload conditions and impaired excitation–contraction coupling under elevated pathological loading. The observed trend closely parallels changes in Cnx43 organization, contractile amplitude, and cardiomyocyte structural organization, supporting a coordinated preload‐dependent regulation of functional maturation‐associated phenotypes.

### 2.6 Mechanical Preload Drives Distinct Transcriptomic Remodeling in hiPSC‐CMs

Transcriptomic analysis demonstrated that cyclic mechanical preload induces distinct gene expression changes in hiPSC‐CMs. At 5 mmHg, we identified 839 genes that were differentially expressed relative to no preload conditions, including 443 upregulated and 396 downregulated genes (Figure 6A). Upregulated transcripts included key cardiac transcription factors and differentiation markers, such as NKX2.5 and HEY2, as well as genes involved in ion channel function (KCNJ2), contractility (S100A4), and extracellular matrix remodeling (FGF21, DPT, and ITGA10), indicating structural reorganization and maturation‐associated transcriptional shifts. At 10 mmHg, the transcriptional response intensified, with 950 genes upregulated and 290 downregulated (Figure 6B). Notably, genes associated with contractile function and sarcomeric stability (CRYAB), cell–matrix force transmission (ITGA7), and ECM integrity (EMILIN2) were upregulated. Developmental regulators such as BMP2 were also elevated, collectively suggesting progression toward a more mature transcriptional profile under moderate physiological loading. In contrast, pathological preload (15 mmHg) triggered extensive transcriptional dysregulation, with 5279 differentially expressed genes (2265 upregulated, 3014 downregulated; Figure 6C). This included significant downregulation of key cardiogenic and mechanoresponsive regulators (NOTCH1, NOTCH3, HEY2, BMP4, BMP10, FZD9), and integrins (ITGA8, ITGA9, and ITGA11). Concurrently, stress‐related and pro‐apoptotic genes such as S100A9 and AIFM2, were upregulated, indicating activation of stress‐adaptive and pathological signaling pathways. Overall, these transcriptomic changes highlight preload‐dependent regulation of gene networks linked to cardiomyocyte differentiation, contractility, ion transport, cytoskeletal remodeling, sarcomere assembly, and structural integrity.

**FIGURE 6:**
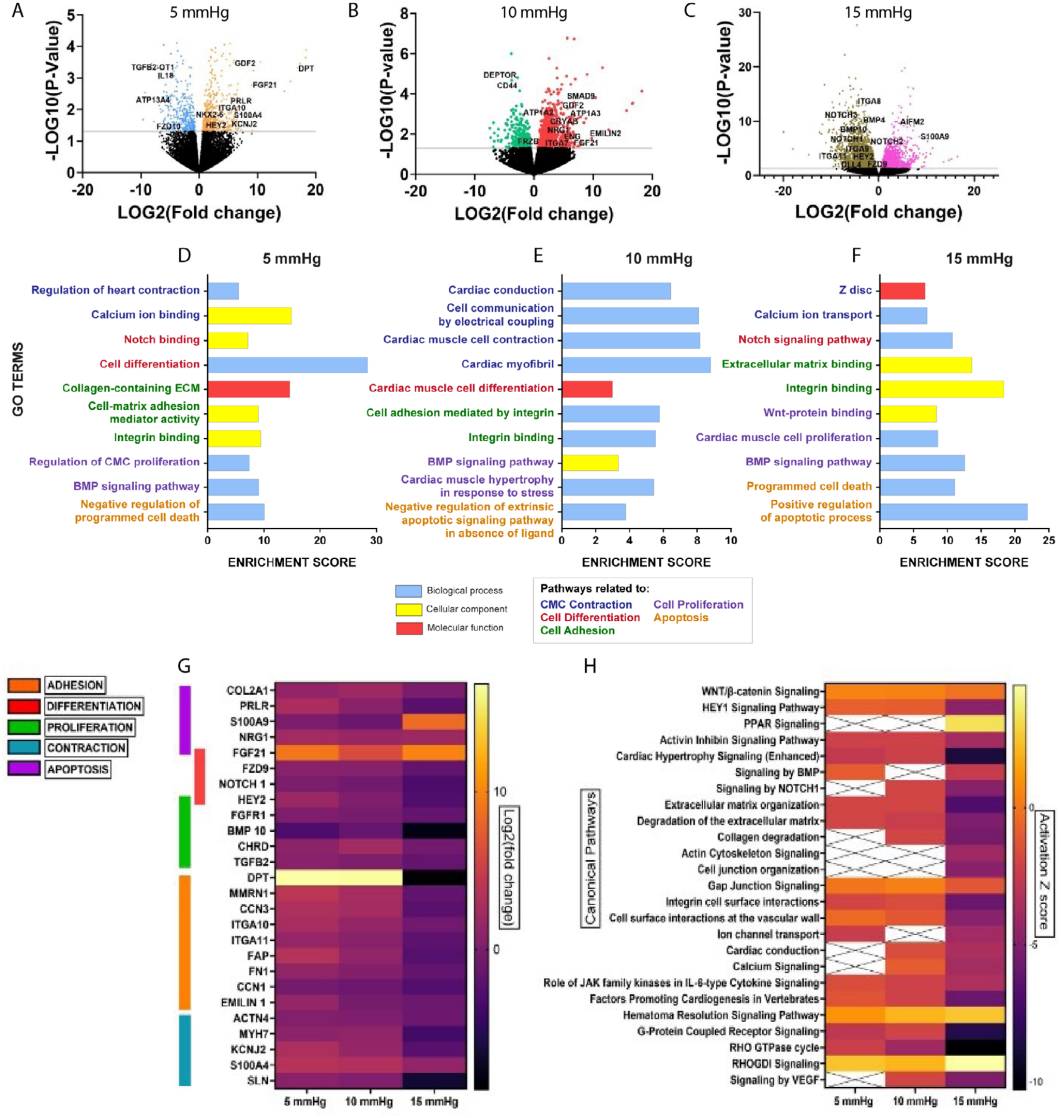
Transcriptomics analysis of hiPSC‐CMs shows differential gene expression in response to changes in preload. Volcano plots showing differential gene expression at (A) 5, (B) 10, and (C) 15 mmHg preload, compared with no preload (0 mmHg). (D–F) GO pathway enrichment analysis across various preload conditions, highlighting pathways associated with cardiomyocyte contraction, cell differentiation, adhesion, proliferation, and apoptosis. (G) Differentially expressed genes identified through GO enrichment, categorized by functional association with adhesion, differentiation, proliferation, contraction and apoptosis. (H) KEGG pathway activation Z‐scores showing the relative activity of key signaling pathways under different preloading conditions. Differential expression was determined using DESeq2 with Benjamini–Hochberg correction (FDR < 0.05). Volcano plots and pathway analyses reflect FDR‐adjusted thresholds.

To investigate preload‐dependent transcriptional responses, we performed differential gene expression analysis using DESeq2 with Benjamini–Hochberg correction for multiple comparisons (FDR < 0.05). Across preload conditions, cyclic mechanical stimulation induced magnitude‐dependent remodeling of gene expression profiles in hiPSC‐CMs. At 5 mmHg, GO enrichment analysis revealed activation of pathways associated with CM differentiation, calcium ion binding, collagen‐containing extracellular matrix, and negative regulation of apoptosis, suggesting transcriptional activation of pathways promoting cardiac lineage commitment and survival (Figure 6D). Upregulated genes (FDR < 0.05) included transcription factors and differentiation regulators (FGF1, FGF21, GDF2, HEY2, and NKX2.5), calcium‐binding protein S100A4, ECM‐associated genes (CCN3, DPT, EMILIN1), and pro‐survival markers (FGF21, PRLR), consistent with enhanced structural organization and cell survival signaling. At 10 mmHg, transcriptional changes reflected increased organization of contractile and electrophysiological machinery (Figure 6E), with enrichment in pathways related to myofibril assembly, electrical coupling, integrin‐mediated adhesion, and BMP signaling. Upregulated genes (FDR < 0.05) included contractile and sarcoma‐associated proteins (CRYAB, MYBPC3, MYH7B, MYL2), membrane ion transport subunits (ATP1A1, ATP1A2, ATP1A3, ATP1B1), adhesion‐related proteins (ITGA7, EMILIN2), and BMP pathway regulators (SMAD9, ENG, CHRD, GDF2), consistent with progression toward a more structurally and functionally organized cardiomyocyte phenotype. In contrast, at 15 mmHg, transcriptomic signatures shifted toward catabolic and stress‐related responses, including enriched pathways (FDR < 0.05) for positive regulation of apoptosis, integrin binding, BMP signaling, and NOTCH signaling (Figure 6F). Upregulated genes included stress‐associated and pro‐apoptotic markers (AIFM2, G0S2, and S100A9), while several mechanoresponsive and adhesion‐related genes (CCN4, ITGA4, ITGA8, ITGA9, ITGA11), as well as BMP (BMP4, BMP10, TGFBR3), and NOTCH pathway components (NOTCH1, NOTCH2, NOTCH3, DLL4, HEY2) were significantly downregulated (FDR < 0.05), consistent with stress‐adaptive transcriptional remodeling observed under elevated mechanical loading conditions.

To investigate the transcriptional response to preload, we analyzed the gene expression patterns associated with cell adhesion, differentiation, proliferation, CM contractility, and apoptosis across different preload conditions (Figure 6G). Genes involved in ECM remodeling (DPT, MMRN1, CCN3 for collagen‐rich ECM and ITGA10, ITGA11, FAP, FN1 for integrin‐mediated adhesion) were increasingly upregulated under 5 and 10 mmHg preload, consistent with increased cell‐cell and cell‐matrix interactions, and ECM stabilization. Conversely, at 15 mmHg, these genes were downregulated, consistent with transcriptional signatures of maladaptive remodeling, matrix degradation, and pro‐apoptotic signaling observed in cardiac disease models [37, 38, 39, 40, 41]. Similarly, structural and functional genes such as MYH7 (sarcomeric Z‐disc), KCNJ2 (potassium channel involved in repolarization), and SLN (calcium binding and transport) were upregulated at 5 and 10 mmHg, but downregulated at 15 mmHg.

Pathway‐level enrichment analysis showed preload‐dependent activation of multiple signaling pathways critical for CM structure and function. Canonical pathways associated with cardiac development and maturation, including Wnt/β‐catenin, Hey1, and NOTCH1 signaling, were upregulated under moderate preloads (5–10 mmHg) but repressed at 15 mmHg, indicating loss of mechanosensitive developmental signaling under pathological load (Figure 6H, Figure S3A). Similarly gap junction signaling and ion channel transport, essential for electromechanical coupling, were both upregulated at 5 and 10 mmHg, but repressed at 15 mmHg.

Pathways involved in structural integrity, such as actin cytoskeleton signaling and cell junction organization, and integrin‐ECM interactions, were progressively downregulated with increasing preload. Notably, PPAR signaling, which regulates metabolic and oxidative stress responses [42], was either stable or was mildly upregulated at moderate preloads but downregulated at pathological preloads (see also Figure S3A). Moreover, Rho GTPase signaling, a cytoskeletal regulatory pathway with cardioprotective roles in heart failure [43], was also downregulated at 15 mmHg. This, coupled with suppression of BMP signaling, extracellular matrix organization and calcium signaling (Figure S3B), suggests that excessive mechanical load induces a shift toward catabolic, stress‐adaptive transcriptomic profile. Furthermore, pathways implicated in cardiac conduction, cell‐cell interaction, and sarcomeric organization were similarly disrupted at 15 mmHg, reflecting stress‐responsive pathway alterations commonly associated with pathological mechanical loading.

## 3 Discussion

The bioreactor developed in this study addresses limitations of existing systems by enabling physiologically calibrated preload application that generates curvature‐induced, multiaxial strain patterns at the cardiomyocyte–substrate interface. Strain patterns correlate with clinically reported end‐diastolic pressure ranges (5–15 mmHg) associated with developmental, adult, and pathological conditions. The average membrane strain values generated within this pressure range (≈9%–15%) fall within the lower end of reported ventricular longitudinal and circumferential strain magnitudes (≈15%–25% shortening) observed during physiological cardiac loading [44, 45, 46]. In contrast, radial strain associated with ventricular wall thickening typically reaches higher magnitudes [45] (≈35%–60%), and is not explicitly modeled in this membrane‐based configuration. Accordingly, our platform approximates preload‐induced fiber‐level mechanical strain while not reproducing full three‐dimensional chamber mechanics or transmural strain heterogeneity.

Our findings reveal that cyclic stimulation under low mechanical preload (≤5 mmHg) activates key developmental signaling pathways, including NOTCH1 and Wnt/β‐catenin, consistent with their established roles in left‐ and right‐sided cardiac morphogenesis, respectively [47]. This activation aligns well with previous studies demonstrating that these pathways transduce mechanical cues into coordinated biochemical and electrophysiological programs that drive early maturation‐associated remodeling [48, 49, 50]. In contrast, exposure to high‐preload conditions (15 mmHg) markedly suppressed these pathways, mirroring dysregulation associated with sustained pathological preload [51, 52, 53].

At physiological preload (10 mmHg), hiPSC‐CMs exhibited anisotropic alignment and enhanced sarcomeric organization, including elongated morphology, more defined Z‐band formation, and improved intercellular connectivity, consistent with progression toward a more mature structural phenotype. Transcriptomic profiles reflected upregulation of ion channel and sarcomeric genes, collectively supporting structural and electrophysiological maturation. Functionally, this was reflected in synchronized and robust contractile activity resembling that of healthy cardiomyocytes with increased contraction amplitude and more compact waveform peaks indicative of improved maturation.

At a pathological preload of 15 mmHg, our bioreactor model reproduced key phenotypic and transcriptomic signatures consistent with cardiomyocyte responses reported under elevated end‐diastolic pressures associated with mechanical overload and heart failure. These changes included disrupted intercellular communication, downregulation of ion channel expression, loss of electromechanical synchrony, hallmarks of maladaptive remodeling reported in various heart failure models [54, 55]. Concurrently, several pro‐survival signaling pathways, such as JAK/STAT, PI3K/Akt, MAPK, were upregulated [56, 57, 58], suggesting a compensatory response to sustained mechanical stress. However, this response was accompanied by enrichment of stress‐associated and apoptosis‐related transcriptional programs, reflecting an imbalance between adaptive stress signaling and maladaptive remodeling associated with progressive myocardial dysfunction.

By applying physiologically and pathologically relevant preload pressure ranges in vitro, our bioreactor provides a powerful platform to study cardiac mechanobiology and evaluate therapeutic responses under defined mechanical conditions. Current studies investigating the biomechanical environment of cardiac cells generally employ planar dynamic stretch along one [13, 59, 60, 61] or more [62, 63] axes in two dimensional configurations, or apply static deformation within three‐dimensional matrices [64, 65, 66]. Commercially available planar stretch systems (e.g. FlexCell, Strex) utilize uniaxial or equibiaxial strain on flexible substrates under cyclic loading, typically reporting mechanical input as prescribed percent strain or actuator parameters. Beyond planar stretch approaches, several membrane‐based platforms use pneumatic actuation to bulge suspended elastomeric diaphragms into non‐planar geometries [67], enabling cardiomyocyte stimulation across spatially varying strain regions or inference of contractile forces from substrate deflection [68].

In these systems, mechanical loading is typically specified as target strain magnitude without explicit link to clinically interpretable end‐diastolic preload pressures. In contrast, our platform anchors cyclic actuation to physiologically defined preload magnitudes (in mmHg) and reconstructs membrane geometry to generate spatial pressure–strain maps that directly relate applied pressure to the local multiaxial loading state at the cardiomyocyte–substrate interface. Our system further permits tunable control of stimulation magnitude, frequency, and duration; parameters known to influence cardiomyocyte structural and functional adaptation through mechanotransduction pathways associated with sarcomere organization, force generation, and maturation [12, 69]. This approach may facilitate patient‐specific disease modeling, precision drug testing, and mechanistically guided therapeutic development that leverages the intrinsic mechanosensitivity of cardiomyocytes.

A comparative benchmarking summary of representative cardiac mechanostimulation platforms is provided in Table S1. Relative to existing pneumatic membrane stretch systems, the present platform combines pressure‐calibrated loading, spatial strain reconstruction, parallelized actuation capability, optical accessibility for high‐resolution imaging, and compatibility with downstream molecular assays within a single experimental framework.

In adherent cell systems, substrate deformation governs cellular mechanical loading, as mechanical cues are transmitted through the deformable substrate to the cell‐substrate interface [70, 71]. Therefore, membrane strain provides a physically grounded proxy for cellular loading; however, direct measurement of subcellular deformation or cell‐generated forces (e.g., via traction force microscopy) was not performed in this study, and would further refine characterization of cell–substrate mechanical coupling.

Here, preload is interpreted as a pressure‐defined mechanical input that induces cardiomyocyte stretch at the cell–substrate interface, rather than direct replication of ventricular chamber mechanics. Our system models preload‐associated mechanical signaling within a controlled in vitro environment, rather than full myocardial loading physiology. The applied pressure range (5–15 mmHg) corresponds to clinically reported end‐diastolic pressures, and the resulting membrane strain magnitudes (≈9%–15%) fall within the lower range of reported myocardial longitudinal and circumferential strain values measured in vivo (e.g., global longitudinal strain ≈12%–22%, including pathological cases) [44, 45, 46]. This comparison is made at the level of strain magnitude and does not imply replication of full myocardial loading conditions.

Although elevated preload (15 mmHg) induced robust stress‐associated transcriptional remodeling consistent with mechanical overload, several limitations should be acknowledged. hiPSC‐CMs do not fully replicate the cellular heterogeneity of native myocardium, as approximately 80% of the cells in our study were cardiomyocytes, with relatively few non‐myocyte populations such as fibroblasts and immune cells that contribute to extracellular matrix remodeling and inflammatory signaling in cardiac disease. In addition, all experiments were performed using a single hiPSC‐CM line, and therefore donor‐ or clone‐dependent variability in mechanotranscriptional responses was not evaluated. Moreover, the short‐term cyclic loading of a two‐dimensional monolayer does not recapitulate the chronic, multicellular, and chamber‐level structural adaptations that characterize clinical cardiomyopathies. Rather than modeling a specific cardiomyopathy subtype, this platform reproduces selected mechanosensitive and stress‐responsive transcriptional pathways triggered by increased preload, providing a controlled framework to study early mechano‐adaptive signaling responses.

## 4 Experimental Section

### 4.1 Bioreactor Design and Fabrication

The bioreactor was designed using Autodesk Fusion 360 and fabricated via SLA 3D printing (Formlabs Form 3). It comprises three primary components (base, top, and cap) assembled into a compact unit with two reservoirs separated by a thin, flexible silicone membrane (0.3 mm thick) (Figure 1A). The top reservoir houses a cell culture well, while the hermetically sealed bottom reservoir functions as a pressure chamber, pneumatically deflecting the membrane to mimic the cyclic mechanical motion of the cardiac tissue (Figure 1A). The 3″ × 2″ baseplate includes a luer‐lock inlet and sealed outlet for pressure control. A pump modulates air pressure within the bottom reservoir, which deflects the membrane upward into the tissue culture well, thereby generating mechanical strain. The tissue culture well, located centrally on the baseplate, was circular with an 18 mm diameter. The membrane was secured between the base and top components with an O‐ring (7/8″ inner diameter) surrounding the culture well, creating a leak‐proof seal between the two reservoirs. The cap, with an integrated top coverslip, was used to cover the culture well to prevent contamination, observation windows (round coverslips) in the cap and base allow for real‐time imaging of cultured cells at magnifications up to 40×. This bioreactor facilitates the application of 3D cyclic mechanical strain to cardiomyocytes cultured on the membrane, supporting continuous imaging and monitoring for up to 72 h. Moreover, it provides convenient access to the cell culture for proteomic, metabolomic, and transcriptomic analyses.

### 4.2 Pneumatic System and Characterization

To study the effects of cyclic mechanical strain on cardiomyocytes, a custom‐built pneumatic system was developed, consisting of a syringe driven by a linear actuator controlled via Raspberry Pi (Figure 1B). The actuator cyclically displaced the syringe plunger to generate controlled pressure waveforms applied to the bioreactor chamber. An inline pressure sensor provided real‐time monitoring and validation of applied pressures. Custom software was developed to generate sinusoidal pressure waveforms at 1 Hz, approximating resting adult heart rate. Developmental and age‐dependent heart rate variations were not specifically modeled. The setup enabled simultaneous operation of multiple bioreactors through a parallel manifold configuration, ensuring equal transmission of cyclic pressures across all bioreactors (Figure 1C). Each manifold port could be independently controlled to operate one or more bioreactors as needed.

### 4.3 Cyclic Preload Application

Bioreactors were connected to the custom‐built pneumatic system for cyclic mechanical stimulation. Control samples were connected to the same manifold, but isolated with closed valves, maintaining identical incubation conditions without pressure exposure. Fresh culture media was added prior to mechanical stimulation. Cyclic preload pressures of 5, 10, and 15 mmHg were applied for 3 h per day over 3 consecutive days, followed by media exchange. On Day 4, bioreactors were rested without stimulation, and membranes were harvested on Day 5 for analysis. Each membrane was sectioned into three equal pie‐shaped segments for live‐dead assays, immunofluorescence staining and transcriptomics analysis. For contractility analysis, intact membranes were imaged prior to sectioning.

### 4.4 Membrane Strain Quantification Under Preload

To quantify membrane strain, fiduciary markers were patterned onto the silicone substrate using microcontact printing in a 7 × 8 array of 2 × 2 mm squares, spaced 1 mm apart edge‐to‐edge. Fluorescent polystyrene beads (≈10 µm) were transferred onto the membrane using a PDMS stamp, ensuring a uniform marker distribution for accurate strain analysis. The bioreactor was mounted on an inverted microscope (Nikon Eclipse Ti2E), and the patterned membrane was imaged under applied pressures ranging from 0 to 15 mmHg. An automated focusing algorithm, integrated with a motorized XYZ positioning stage, determined lateral and axial positions of the membrane at the patterned locations. These spatial coordinates were used to reconstruct the three‐dimensional membrane surface at each pressure by fitting a continuous surface model to the deformation profile. Strain fields were computed from the reconstructed geometry, generating spatial strain maps for each pressure condition. Representative membrane strain was quantified using line‐averaged engineering strain across the membrane diameter. Average strain values were derived from three independent deformation measurements (*n*  =  3). Local cellular strain was inferred from global membrane deformation rather than measured directly at the subcellular level. Because cardiomyocytes form adherent monolayers on the deformable substrate, in‐plane membrane strain provides a spatially resolved proxy for the mechanical loading transmitted to the cell–substrate interface. A complementary shape‐prescribed mechanical model incorporating compliant boundary representation was developed to relate experimentally measured center deflection to representative membrane strain and to evaluate sensitivity to kinematic and constitutive assumptions (Section S3).

### 4.5 Cell Preparation and Culture

Cardiomyocytes were generated from a single hiPSC line through three independent differentiation experiments performed on separate days. These independent differentiation batches served as biological replicates (*N*  =  3), from which quantitative imaging and functional measurements were obtained. Cells were cultured on Matrigel‐coated 6‐well plates (Corning) until ≈90% confluency. Prior to differentiation, cultures were gently dissociated using 0.5 mM EDTA in PBS (ThermoFisher) for up to 4 min. Cardiomyocyte differentiation was initiated by culturing cells in RPMI 1640 (Gibco) supplemented with B27 minus insulin (Gibco) and 6 µM CHIR‐99021 (Selleck Chemical) for 2 days. This was followed by 1 day in RPMI B27 minus. On Day 3, Wnt signaling was inhibited using 5 µM IWR‐1 (Sigma Aldrich) in RPMI B27 minus for 2 days. Cultures were then maintained in RPMI 27 minus for an additional 2 days without the inhibitor. Starting on Day 7, cells were maintained in RPMI B27 plus (Gibco), with media changes every 2 days until spontaneously contractions were observed (typically by Day 15). Between Days 15–20, cardiomyocytes were transferred to the bioreactors for mechanical stimulation experiments (Figure 2A).

### 4.6 Bioreactor Assembly and Cell Seeding

Upon differentiation, hiPSCs‐CMs were seeded onto the bioreactors (Figure 2B). Biocompatible silicone membranes were cut into 1.25″ discs using a hollow punch and sterilized prior to use. Bioreactor components were sterilized by immersion in 70% ethanol for 10–15 mins, followed by overnight UV exposure. Membranes were secured between the bioreactor base and the tissue culture chamber, with an O‐ring ensuring a leak‐proof seal around the chamber. Following assembly, bioreactors were tested with the custom pneumatic system to ensure proper sealing and absence of leaks. To promote cell adhesion, 1 mL of diluted Matrigel (0.1 mg ml^−1^) was applied to each membrane 24 h before seeding. Approximately 1 × 10^6^ hiPSC‐CMs were added per bioreactor and allowed to adhere for 48 h. Cultures with <75% confluency were discarded. For bioreactor cultures with >75% confluency, cyclic preload pressures of 5, 10, and 15 mmHg were applied for 3 h/day over 3 days. Culture media was replaced with RPMI B27 plus antibiotics (ThermoFisher) immediately before stimulation.

### 4.7 Live/Dead Assay and Cell Viability

Cell viability was assessed using the Live/Dead Viability/Cytotoxicity Kit (ThermoFisher) following the manufacturer's protocol with minor modifications. After removing the culture medium, membranes were incubated with the dye solutions for ≈30 min, followed by a PBS wash. Nuclei were counterstained with Hoechst 33342 (Life Technologies) for 15 mins at room temperature, followed by a final PBS wash. Samples were imaged by confocal microscopy. Live cells were imaged in the green channel (Ex/Em: 475/523 nm), and nuclei were imaged in the blue channel (Ex/Em: 350/454 nm). Image analysis was performed in ImageJ. Fluorescence images were converted to 16‐bit, background subtracted, thresholded, and binarized. Cell segmentation was performed using the watershed algorithm. Finally, cell counts were obtained using the ‘Analyze Particles’ function. Viability was expressed as a percentage of calcein positive cells relative to total Hoechst‐stained nuclei.

### 4.8 Immunofluorescence (IF) Staining

To assess cardiomyocyte differentiation, structural organization, and connectivity following exposure to cyclic mechanical strain, immunofluorescence (IF) staining was performed for NKX2.5, α‐actinin, and connexin‐43, respectively. Cells adherent to bioreactor membranes were fixed in 4% paraformaldehyde (PFA) for 15 min at room temperature, followed by a triple PBS wash (5 min each). Cells were permeabilized using 300 µl of methanol for 2 min at room temperature. Membranes were then blocked with 3% BSA in PBS for 2 h at room temperature. Membranes were initially incubated overnight at 4°C with primary antibodies against NKX2.5 (Cell Signaling Technology) (1:1600), α‐actinin (Sigma Aldrich) (1:800) and connexin‐43 (Sigma Aldrich) (1:100), all diluted in PBS. After three PBS washes (5 min each), membranes were incubated with secondary antibodies (1:500 in PBS) for 2 h at room temperature in the dark: anti‐rabbit IgG (A21428, ThermoFisher) for NKX2.5, anti‐mouse IgG (715‐175‐151, Jackson ImmunoResearch) for α‐actinin and anti‐rabbit IgG for connexin‐43. Following this incubation, membranes were washed three times with PBS. Nuclei were stained with Hoechst 33342 (Invitrogen H3570, 1:500 in PBS) for 5 min at room temperature and rinsed twice with PBS. Confocal images were acquired using a Nikon A1R HD microscope (20x magnification) with NIS‐Elements AR (v5.41.02, 64‐bit). Imaging was performed using the following fluorescence channels: 425/475 nm for blue, 500/550 nm for green, 570/620 nm for red. Quantitative image analysis was performed using ImageJ.

### 4.9 Analysis of Cell Size and Aspect Ratio

Fluorescence images were acquired at four radial positions (90° apart) across the membrane using a confocal microscope (Nikon A1R HD) at 20x magnification. Cells were co‐stained with α‐actinin to visualize cell boundaries and Hoechst to label nuclei. Image analysis was performed in ImageJ. For each identified cell, the longest linear dimension was recorded as the long axis length and used to approximate overall cell size. The perpendicular shortest dimension was recorded as the short axis length. Aspect ratio was calculated as the ratio of long axis to short axis length.

### 4.10 Quantification of Cell Density and Cardiomyocyte Differentiation

Cell density was quantified using Hoechst‐stained nuclei from membrane‐adherent cells. The number of Hoechst‐positive nuclei within each field of view (FOV) was determined using ImageJ, and cell density was calculated as nuclei count per FOV area. Cardiomyocyte differentiation was assessed by immunofluorescence staining for NKX2.5. The number of NKX2.5‐positive cells was divided by the number of Hoechst‐positive nuclei to determine the percentage of cardiomyocytes.

### 4.11 Connexin‐43 Localization and Density

Connexin‐43 (Cnx43) localization and expression were analyzed to evaluate cardiomyocyte intercellular connectivity. Confocal Z‐stacks were acquired to capture Cnx43 immunofluorescence at 0.5 µm intervals across the full thickness of the cardiomyocyte layer using identical imaging settings for all experimental groups. Maximum intensity Z‐projections were generated in ImageJ for quantitative analysis. Cell‐cell junctions were manually outlined using the freehand selection tool, and mean fluorescence intensity was measured following background subtraction from each ROI. Total Cnx43 expression was quantified as corrected mean fluorescence intensity within the analyzed field of view. To assess junctional localization independently of total expression level, the fraction of Cnx43 fluorescence localized at intercellular interfaces was calculated by normalizing junctional Cnx43 intensity to total Cnx43 fluorescence within the same image. The percentage of cells with peripheral Cnx43 localization was calculated by dividing the number of cells displaying junction‐associated Cnx43 staining by the total number of Hoechst‐positive nuclei within each field of view.

### 4.12 Evaluating Cardiomyocyte Contractility

Following 72 h of exposure to cyclic strain, membranes were transferred to Mattek dishes with fresh RMPI/B27 medium and incubated for 2 h at 37°C for recovery. Spontaneous cardiomyocyte contractions were recorded at 20x magnification using a Nikon TiE microscope with an ANDOR Neo/Zyla camera and NIS Elements AR (4.50.00 64‐bit). Movies were captured at 50 fps for 15–20 s under physiological conditions (temperature, CO_2_, and humidity control). Contractile parameters including beat frequency, contraction amplitude, peak time, time to peak contraction, and relaxation time were quantified using Myocyter (v. 1.0), an open source ImageJ macro that derives metrics from frame‐to‐frame pixel intensity changes [72].

### 4.13 Calcium Imaging

Approximately 40–50 ROIs were analyzed per video per video for samples conditioned under cyclic preload pressures ranging from 0–15 mmHg. Calcium transient traces and peak detection outputs were visually inspected to verify accurate identification of calcium events, and recordings with substantial segmentation or peak‐detection artifacts were excluded from analysis. Quantitative measurements obtained from each biological replicate were averaged prior to statistical comparison between experimental groups. Statistical significance was assessed using one‐way ANOVA followed by Tukey's multiple‐comparison test.

### 4.14 Transcriptomics Analysis

mRNA was extracted using the Quick‐RNA Miniprep kit (R1054, Zymo Research) following the manufacturer's protocol. RNA was eluted with 50 µL of nuclease‐free water directly added to the column matrix, followed by centrifugation into a clean collection tube. The elute containing purified mRNA was placed on ice. mRNA concentration and purity were determined using a NANODROP 2000 Spectrophotometer (ThermoFisher). Total RNA quality and integrity were assessed by agarose gel electrophoresis (Novogene Corporation Inc). RNA‐seq libraries were prepared using the Illumina TruSeq protocol and paired‐end sequencing was performed on the Illumina HWI‐ST1276 platform. Sequence reads were aligned to the human reference genome using STAR v2.5. Gene‐level read counts were generated using HTSeq v0.6.1. Expression levels were calculated as fragments per kilobase of transcript per million mapped reads (FPKM) for visualization and relative expression comparisons, but were not used for statistical testing. Differential expression analysis was conducted in Partek Flow using DESeq2 with raw read counts as input. Library size normalization was performed using DESeq2's median‐of‐ratios method. Statistical significance was assessed using the Wald test, and p‐values were adjusted for multiple comparisons using the Benjamini–Hochberg false discovery rate (FDR). Genes with adjusted p‐values (FDR < 0.05) were considered significantly differentially expressed. Gene set enrichment analysis (GSEA) and pathway‐level analyses were performed using Ingenuity Pathway Analysis (IPA) with KEGG annotations. Pathways were considered significantly enriched at Benjamini–Hochberg adjusted ***p*** < 0.05, and activation states were interpreted based on Z‐score thresholds (> +2 for activation, < –2 for inhibition). For GSEA, gene sets meeting standard FDR q‐value thresholds were considered significantly enriched. All samples were derived from the same hiPSC line and processed under identical culture, RNA extraction, library preparation, and sequencing conditions to minimize technical variability.

### 4.15 Statistical Analysis

All quantitative data were presented as mean ± standard deviation (SD) unless otherwise indicated. Biological replicates (***N *** = * *3) represent independent cardiomyocyte differentiation experiments performed from the same hiPSC line on separate days. Quantitative imaging and functional analyses were derived from multiple cells, image fields, regions of interest, or calcium transients collected from each biological replicate. For statistical analysis, measurements obtained within each biological replicate were averaged prior to comparison between experimental groups. For membrane characterization experiments (Figure 1), ***n*** denotes independent deformation measurements. Statistical analyses were performed using GraphPad Prism (v10.2.2). For comparisons among preload conditions, one‐way ANOVA with two‐sided testing was used (***α *** = * *0.05), followed by Tukey's multiple‐comparison test. Homogeneity of variance was assessed using Bartlett's and Brown–Forsythe tests where appropriate. Statistical significance was defined as ***p*** < 0.05 and exact p‐values were provided in the corresponding figure legends. Statistical methods for transcriptomic analysis, including multiple‐testing correction using the Benjamini–Hochberg FDR approach, were described in the Transcriptomics analysis section.

## Author Contributions

**H.M**. contributed to methodology, investigation, and visualization, and participated in writing – original draft. **G.J**. contributed to methodology, investigation, and visualization. **J.P**. contributed to methodology, investigation, and visualization. **H.P**. contributed to methodology, investigation, and visualization. **G.H**. contributed to methodology and investigation. **J.J**. contributed to methodology and investigation. **R.S**. contributed to methodology and investigation. **A.G**. contributed to methodology and investigation. **A.S**. contributed to conceptualization, methodology, supervision, and writing – original draft and review & editing. **M.T**. contributed to conceptualization, methodology, supervision, and writing – original draft and review & editing. **L.K**. contributed to conceptualization, supervision, and writing – original draft and review & editing.

## Conflicts of Interest

The authors declare no conflicts of interest.

## Supporting information




**Supporting File 1**: advs76483‐sup‐0001‐SuppMat.docx.

**Supporting File 2**: advs76483‐sup‐0002‐MovieS1‐S4.zip.

## Data Availability

All codes and algorithms will be uploaded to the GitHub cloud server for access. The transcriptomics data will be uploaded to NIH datahub.
