# Supplementary material for: Cyclic Mechanical Loading of Cardiomyocytes via Pressure‐Driven Non‐Planar Membrane Deformation in a Bioreactor System
Source: Adv Sci (Weinh). 2026 Jul 13;13(55):e23820. doi: 10.1002/advs.202523820 (PMC13360101; doi:10.1002/advs.202523820)
Supplement: Supplementary file 1 [file ADVS-13-e23820-s002.docx]

Supporting Information

Cyclic Mechanical Loading of Cardiomyocytes via Pressure-driven Non-Planar Membrane Deformation in a Bioreactor System

Haris Mansoor, Gabrielle Juul, Jil Patel, Heran Pradhan, Gram Hepner, Jackson Jewell, Mark Bardin, Ryan Slusser, Abigail Gezer, Leda Klouda, Melikhan Tanyeri*, Anita Saraf*

S1. Sarcomeric architecture is enhanced by physiological preload and disrupted by pathological strain

To further evaluate the structural maturation of hiPSC-CMs under different mechanical loading conditions, we analyzed α-actinin immunostaining to assess sarcomeric organization (**Figure S1**). While cells under static conditions exhibited disorganized, punctate sarcomeric patterns, cyclic preload at 5 and 10 mmHg promoted clear Z-band formation and anisotropic alignment, consistent with improved structural maturation. In contrast, excessive preload at 15 mmHg led to disrupted sarcomeric architecture.

Figure S1. Effect of cyclic preload on morphology of hiPSC-CMs. Following α-actinin staining, sarcomeres are visible under 0, 5, and 10 mmHg preloading conditions. Most prominent and abundant sarcomeres with directionality are observed at 10 mmHg. Loss of cellular architecture and sarcomeres is observed at 15mmHg.

Figure S2. Representative waveforms of beating cardiomyocytes. Myocyter analysis of brightfield videos of spontaneously beating hiPSC-CMs demonstrate that as preloading conditions increased from (A) 0 mmHg to (B) 5 mmHg, then to 10 mmHg (Fig. 5F, main text), contraction waveform width and the beating frequency noticeably decreased. (C) At 15 mmHg, the beats were irregular in width, amplitude, and morphology.

Figure S3. Preload-Dependent Regulation of Cytoskeletal and Mechanosensitive Signaling Pathways. (A) Number of downregulated genes in each KEGG pathway. (B) Venn diagram representing differentially expressed genes in each preload condition as compared to control.

S2. Comparison of Cardiac Mechanical Loading Platforms with this Bioreactor System

Table S1 summarizes key distinctions between existing cardiac mechanical loading platforms and the present bioreactor system. In contrast to conventional planar stretch systems and many heart-on-chip approaches that define stimulation primarily through actuator settings or prescribed strain, our platform directly links clinically relevant pressure-defined loading to experimentally reconstructed spatial strain fields. The system combines pressure-calibrated cyclic actuation, three-dimensional membrane geometry reconstruction, and multiaxial strain mapping within an imaging-compatible monolayer format that supports downstream molecular and functional analyses. Our bioreactor enables real-time, high-resolution imaging using brightfield, phase-contrast, fluorescence, and confocal microscopy, allowing continuous visualization and analysis of cardiomyocyte behavior during mechanical stimulation. While the platform does not reproduce the full volumetric complexity or cellular heterogeneity of native myocardium, it provides a quantitatively defined and experimentally accessible framework for studying preload-associated cardiomyocyte mechanobiology.

**Table S1**. Comparison of Cardiac Mechanical Loading Platforms and Key Functional Capabilities

| Platform Type | Typical Mechanical Input | Common Limitations | Pressure-Calibration | 3D Geometry Reconstruction | Multiaxial Strain Mapping | Imaging / Downstream Assay Compatibility | Throughput |
| --- | --- | --- | --- | --- | --- | --- | --- |
| Planar Stretch Systems | Uniaxial or equibiaxial membrane elongation (vacuum/mechanical actuation) | Primarily planar deformation; largely uniform strain fields; mechanical input reported as strain or displacement | No | No | Yes | Yes/Yes | Moderate |
| Heart-on-Chip / Microfluidic Platforms | Pneumatic/piezoelectric/thermal/fluidic membrane actuation; sometimes coupled with 3D tissue constructs | Mechanical stimulation defined by actuation parameters; spatial strain mapping often limited | Rare | Limited | Indirect | Variable/Moderate | Low–Moderate |
| Engineered Heart Tissues (EHT) / Microtissues | Anchor- or post-based mechanical loading of scaffolded three-dimensional tissue constructs | Complex workflows; limited optical access; reduced throughput; preload not always pressure-defined | No | Limited | Limited | Limited/Yes | Low |
| This Study | Curvature-driven, out-of-plane membrane deformation anchored to defined end-diastolic pressure profiles | 2D monolayer (not volumetric tissue), limited cellular heterogeneity | **Yes** | **Yes** | **Yes** | **Yes** | **Moderate** |

S3. Mechanical Modeling and Validation of a Pressure-Deflected Circular Silicone Membrane

We developed a mechanical model to estimate deformation and strain in a thin circular silicone membrane subjected to static pressure loading from one side (underneath). The objective of the model was twofold: (i) to relate applied pressure and experimentally measured center deflection to representative membrane strain, and (ii) to provide a physically interpretable basis for comparing the three-dimensional deformation of the pressurized membrane to conventional planar membrane stretching. Particular emphasis was placed on identifying the dominant physical mechanisms governing strain and on evaluating the consistency of the model with experimental strain measurements.

***Membrane geometry, material, and experimental boundary conditions:*** The membrane is a thin liquid silicone rubber (LSR) sheet of nominal thickness 0.3 mm, with material properties typical of medical-grade silicone elastomers (Shore A hardness 40–50). Static pressures between 0 and 15 mmHg are applied through a reservoir beneath the membrane.

The membrane perimeter is constrained using two rigid clamping components in combination with a polymer O-ring (1/16″ cross-section, nominal inner diameter 7/8″). The O-ring prevents gross slipping of the membrane but introduces a compliant constraint rather than a perfectly rigid clamp. Consequently, the experimental boundary condition is intermediate between ideal clamped and simply supported limits, with some redistribution of strain near the membrane edge. Because this boundary compliance cannot be described exactly from geometry alone, it is represented in the model through effective kinematic parameters, as described below.

***Deflection profile and kinematic assumptions:*** For thin membranes undergoing large deflection, membrane strain is governed primarily by geometric nonlinearity once the displacement field is specified [1], whereas uncertainties in boundary compliance (e.g., O-ring constraint) and constitutive parameters can dominate predictions in pressure-driven forward models. Accordingly, shape-based strain estimation is commonly used when deformation can be measured directly and boundary conditions are not perfectly defined, including in experimental mechanics of pressurized membranes/shells and in continuum biomechanics of soft tissues [2, 3]. Here, we therefore employed a shape-prescribed model driven by experimentally measured center deflection to estimate strain from kinematics, enabling robust strain estimation without over-constraining the model with uncertain constitutive properties or boundary assumptions. The membrane was assumed to deform axisymmetrically, with the out-of-plane deflection described by:

$w\left( r \right)=w_{0}\left[ 1 - \left( \frac{r}{a} \right)^{n} \right]$	(Eqn. S1)

where $w_{0}$ is the measured center deflection, $a$ is an effective free-span radius, and $n$ is a shape exponent that controls curvature near the membrane edge.

This functional form provides a compact representation of membrane bulging while allowing flexibility in the representation of edge compliance. The shape exponent $n$ effectively reflects the stiffness of the boundary constraint. A nominal value of $n=3$ was adopted for the primary analysis, as it produced deflection profiles consistent with experimental observations of O-ring-constrained membranes. Values in the range $n=2-5$ yielded similar qualitative strain distributions and comparable average strain levels, indicating that the predicted average strain is not highly sensitive to the precise value of $n$.

Two characteristic diameters define the system: the pressurized reservoir beneath the membrane (~18 mm) and the inner O-ring diameter (~22 mm). To represent the actively deforming region, we adopted an effective membrane radius of $a=9 mm$ based on the reservoir diameter, reflecting that deformation is primarily localized above the pressurized region while the O-ring behaves as a compliant rather than perfectly clamped boundary. This compliant-boundary approximation yielded strain predictions consistent with experimental measurements.

***Strain estimation methods***

Strain was evaluated using multiple modeling approaches to assess the sensitivity of predicted membrane strain to kinematic and constitutive assumptions. Comparing these approaches enables identification of the dominant mechanisms governing membrane deformation under loading and provides bounds on representative strain levels.

***Geometric strain:*** A purely kinematic estimate of strain was obtained by considering the elongation of the membrane induced by out-of-plane deflection. For an axisymmetric membrane, the stretch in the meridional (radial) direction was approximated as:

$\lambda_{\text{g}}=\sqrt{1+\left( \frac{dw}{dr} \right)^{2}}$	(Eqn. S2)

with the corresponding engineering strain given by $\varepsilon_{\text{g}}=\lambda_{\text{g}}-1$. This geometric strain captures the finite rotation and curvature associated with large deflection independently of material constitutive behavior or in-plane stress assumptions.

***Linear elastic membrane strain:*** For comparison, in-plane strains were also computed using von Kármán membrane kinematics, which account for geometric nonlinearity arising from moderate out-of-plane deflection while assuming small elastic strains. Under axisymmetric deformation, the radial and circumferential engineering strains are given by:

$\varepsilon_{\text{r}}=\frac{du}{dr}+\frac{1}{2}\left( \frac{dw}{dr} \right)^{2}$,	$\varepsilon_{\text{θ}}=\frac{u}{r}+\frac{1}{2}\left( \frac{dw}{dr} \right)^{2}$	(Eqn. S3)

where $u\left( r \right)$ is the in-plane radial displacement and $w\left( r \right)$ is the prescribed out-of-plane deflection. This approach captures the leading-order coupling between membrane stretching and bending-induced curvature but neglects higher-order finite-strain effects. While formally valid only for small elastic strains, this approach provides a useful reference and highlights the limitations of linear membrane theory at larger deflections.

***Neo-Hookean finite-deformation strain:*** To account for finite-strain kinematics appropriate for silicone elastomers, in-plane deformation was additionally evaluated using a Neo-Hookean framework. Under axisymmetric deformation, the radial and circumferential stretches were computed from the displacement field as:

$\lambda_{\text{r}}=\sqrt{\left( 1+\frac{du}{dr} \right)^{2}+\left( \frac{dw}{dr} \right)^{2}}$,	$\lambda_{\text{θ}}=\frac{r+u}{r}$	(Eqn. S4)

where $u(r)$and $w(r)$ denote the in-plane and out-of-plane displacements, respectively. Representative strain was then reported using the engineering strain measure $\varepsilon=\lambda-1$. This approach accounts for large rotations and finite stretching while remaining independent of specific material parameters for the purposes of strain estimation and thus provides a physically appropriate comparison for the linear membrane model at larger deflections.

***Strain mapping and averaging***

Strain fields were mapped onto the three-dimensional deflected membrane surface for visualization (**Figure S4**). All models predicted strongly non-uniform strain distributions, with peak strain near the membrane periphery and lower strain near the center.

To enable comparison with one-dimensional membrane stretching experiments, strain was quantified using the line-averaged engineering strain across the membrane diameter:

$$\begin{matrix} \bar{\varepsilon}_{line}=\frac{1}{a}\int_{0}^{a} \varepsilon\left( r \right)dr & (Eqn. S5) \end{matrix}$$

which, under axisymmetric deformation, is equivalent to averaging strain along the full membrane diameter. Unlike area-averaged strain, this metric does not weight deformation by radial position and therefore provides a more direct analog to the average elongation of a material fiber or gauge-length measurement in a flat membrane.

**Fig. S4:** Three-dimensional strain maps of the pressure-deflected membrane predicted using geometric, linear elastic, and Neo-Hookean models. **(A–C)** Geometric engineering strain, **(D–F)** linear elastic membrane strain using von Kármán kinematics, and **(G–I)** Neo-Hookean engineering strain for membrane pressures of 5, 10, and 15 mmHg, respectively. Strain fields were mapped onto the reconstructed three-dimensional membrane geometry using an effective membrane radius of $a=9$mm and shape exponent $n=3$. All models predicted non-uniform strain distributions, with strain localized primarily near the membrane periphery. The geometric and Neo-Hookean models produced similar strain distributions and representative strain levels, indicating that membrane deformation was dominated primarily by geometric effects associated with large deflection and finite rotation, whereas the linear elastic model predicted elevated strain at larger deflections. Color bars indicate engineering strain (unitless) with identical scaling across all panels.

Based on the simulations in Fig. S4, using the nominal parameters $a=9$mm and $n=3$, the predicted line-averaged engineering strains from both the geometric and Neo-Hookean models showed excellent agreement with experimentally measured strain values across the full pressure range studied (5–15 mmHg). Representative results are summarized in **Table S2**.

**Table S2.** Experimentally measured center deflections and corresponding modeled line-averaged strains.

| Pressure (mmHg) | Center deflection (mm) | Average experimental strain (%) | Geometric strain (%) | Neo-Hookean strain (%) |
| --- | --- | --- | --- | --- |
| 5 | 3.15 | 9.8 | 9.8 | 10.1 |
| 10 | 3.59 | 12.0 | 12.3 | 12.6 |
| 15 | 4.08 | 14.5 | 15.4 | 15.8 |

The close correspondence between the two nonlinear models indicates that membrane strain in this regime is governed primarily by geometric effects associated with large deflection and finite rotation, rather than detailed material hyperelasticity. In contrast, linear elastic membrane theory systematically overestimated strain at larger deflections, consistent with its limited range of validity.

Although the agreement between model predictions and experimental measurements is strong, it is contingent on the assumed boundary conditions and kinematic representation of membrane deformation. In particular, the effective radius $a$ and shape exponent $n$ provide indirect representations of the O-ring constraint and are not independently measured quantities. While predicted average strains were relatively insensitive to moderate variation in $n$, additional experimental characterization of displacement and strain near the membrane edge would be required to directly validate these assumptions.

In summary, a shape-prescribed mechanical model incorporating realistic boundary compliance provides an accurate and physically interpretable description of strain in a pressure-deflected silicone membrane. When representative strain is quantified using line-averaged engineering strain, geometric and Neo-Hookean models yield predictions in excellent agreement with experimental measurements, suggesting that geometric considerations dominate membrane strain in this system. At the same time, the model relies on reasonable but idealized representations of boundary conditions, underscoring the importance of further experimental validation in future work.

***FEM-based membrane deflection model***

In addition to the shape-prescribed models described above, a finite element model (FEM) of membrane deformation was developed in COMSOL Multiphysics to evaluate pressure-driven deformation using an explicit constitutive mechanics framework. A geometrically nonlinear plate/shell formulation based on Reissner–Mindlin-type kinematics was used to estimate membrane deflection and in-plane strain under applied pressure loading. Mid-surface membrane strains were computed from displacement gradients according to:

$$\begin{matrix} \gamma_{\alpha\beta}=\frac{1}{2}\left( \frac{\partial u_{\alpha}}{\partial x_{\beta}}+\frac{\partial u_{\beta}}{\partial x_{\alpha}} \right) & (Eqn. S6) \end{matrix}$$

Unlike the shape-prescribed strain estimation approaches, the FEM model explicitly incorporated constitutive material behavior through the Young’s modulus and Poisson’s ratio of the silicone membrane. Boundary conditions were defined such that the membrane rim was fixed while a distributed pressure load corresponding to the applied pneumatic pressure was applied to the lower membrane surface.

The resulting membrane deformation and strain distributions are shown in **Figure S5**. Simulated membrane deflections and average strains demonstrated reasonable agreement with experimental measurements across the applied pressure range (**Table S3**). In particular, FEM-based membrane displacement and strain values closely matched experimentally measured center deflection and average strain values at intermediate and higher pressures.

**Table S3.** Experimental and FEM model for center membrane displacement and average strains.

| Pressure (mmHg) | Center deflection (mm) | FEM displacement (mm) | Average experimental strain (%) | FEM strain (%) |
| --- | --- | --- | --- | --- |
| 5 | 3.15 | 2.63–2.90 | 9.8 | 6.18–7.59 |
| 10 | 3.59 | 3.33–3.68 | 12.0 | 9.99–12.23 |
| 15 | 4.08 | 3.82–4.22 | 14.5 | 13.21–16.15 |

The FEM simulations additionally highlighted the sensitivity of pressure-driven forward models to assumptions regarding boundary compliance, constitutive behavior, and effective clamping conditions. This observation is consistent with the experimentally constrained shape-prescribed modeling framework described above, where strain estimation was derived directly from measured membrane geometry rather than inferred solely from constitutive and boundary assumptions.

Collectively, the geometric, Neo-Hookean, and FEM models yielded comparable representative strain levels, supporting the conclusion that membrane deformation in this regime is governed primarily by large-deflection geometry and finite rotation, while constitutive nonlinearities play a secondary role within the investigated pressure range.

**Fig. S5:** Three-dimensional strain maps of the pressure-deflected membrane predicted based on finite element analysis using COMSOL.

Movie S1.

Brightfield recording of spontaneously beating hiPSC-derived cardiomyocytes within the bioreactor under static conditions (0 mmHg), showing baseline beating frequency and contraction waveform.

Movie S2.

Brightfield recording of spontaneously beating hiPSC-derived cardiomyocytes under 5 mmHg preload.

Movie S3.

Brightfield recording of spontaneously beating hiPSC-derived cardiomyocytes under 10 mmHg preload with reduced beating frequency and increased contraction amplitude.

Movie S4.

Brightfield recording of spontaneously beating hiPSC-derived cardiomyocytes under 15 mmHg preload, showing reduced contraction amplitude and irregular waveform patterns indicative of mechanical stress.

**References**

1.	Timoshenko, S. and S. Woinowsky-Krieger, *Theory of plates and shells*. 2d ed. Engineering societies monographs. 1959, New York,: McGraw-Hill. 580 p.

2.	Vella, D., et al., *The indentation of pressurized elastic shells: from polymeric capsules to yeast cells.* Journal of The Royal Society Interface, 2011. **9**(68): p. 448-455.

3.	Humphrey, J.D., *Review Paper: Continuum biomechanics of soft biological tissues.* Proceedings of the Royal Society A: Mathematical, Physical and Engineering Sciences, 2003. **459**(2029): p. 3-46.
